# Supplementary material for: Assessment of the performance and challenges in the implementation of the test, treat and track (T3) strategy for malaria control among children under-five years in Ghana
Source: PLoS One. 2022 Dec 7;17(12):e0278602. doi: 10.1371/journal.pone.0278602 (PMC9728892; doi:10.1371/journal.pone.0278602)
Supplement: Attachment — Submitted filename: Response to Reviewers.docx [file pone.0278602.s002.docx]

**Response to Reviewers’ Comments**

| **Reviewer** | **Comment** | **Response** |
| --- | --- | --- |
| **Reviewer 1** | This paper addresses an important issue in the implementation of T3 strategy of WHO for malaria. For me the most important finding was that Health Workers do not trust the RDTS. This has also been found in previous studies, for example : 1) The effect of malaria rapid diagnostic tests results on antimicrobial prescription practices of health care workers in Burkina Faso. Bonko MDA, et al. Ann Clin Microbiol Antimicrob. 2019 Jan 28;18(1):5. doi: 10.1186/s12941-019-0304-2 2) Accuracy of a Plasmodium falciparum specific histidine-rich protein 2 rapid diagnostic test in the context of the presence of non-malaria fevers, prior anti-malarial use and seasonal malaria transmission. Kiemde F, et al Malar J. 2017 Jul 20;16(1):294. doi: 10.1186/s12936-017-1941-6 And these papers (or comparable ones should be mentioned in the introduction or discussion. | Thank you for your comment and for suggesting these two important papers that had a similar challenge of clinicians not trusting the results of RDTs as found in our study.  As suggested we have now mentioned these two papers in our discussion. This can be found on lines 448-449 and reads as “Studies in Burkina Faso have shown that clinicians do not trust the results of RDTs as reported in our study [26, 27]” |
|  | It is noted that the data were collected about 5 years ago. How valid are these nowadays? Why did it take so long to prepare a manuscript. Please explain. | Thank you for your comment. We acknowledge that the data were collected 5 years ago but we believe that the findings are still valid since no major intervention has been implemented by the Ghana Malaria Control Programme to address the gaps identified in the regions the study was conducted.  The reason for which it took so long for the manuscript to be prepared was because the data collected was to be used for a manuscript writing workshop which was to be used as a learning process for students of the School of Public Health of the University of Health and Allied Sciences where the Principal Investigator is a Professor. However, the manuscript writing workshop could not come off due to some interruptions in the School’s academic calendar which was further worsened by the COVID 19 pandemic and Schools were closed down. The workshop could not be organized via zoom since most of these students lived in remote areas where internet connectivity was poor hence the delay in the manuscript preparation.  We have now included this in our discussion and it can be found on lines 459-462 and reads “Although data for this study was collected about 5 years ago, the findings are still relevant in informing interventions to improve adherence to the T3 strategy in health facilities in Ghana since to the best of our knowledge no specific intervention has been implemented in the regions the study was conducted to improve adherence to the strategy.” |
|  | It would be very useful to discuss the type of RDT(s) that have been used in the facilities for the diagnosis of malaria. Are these notorious in providing incorrect diagnosis? | Thank you for your comment.  We have now included in our discussion the type of RDT kits used in the health facilities and mentioned studies that have evaluated this test kit and found it to have satisfactory agreement with traditional methods of testing like microscopy and PCR.  This revision is found on lines 449-455 and reads “In Ghana the RDT kit mainly supplied to health facilities by the National Malaria Control Programme is the SD BIOLINE Malaria Ag P.F (HRP2/pLDH) manufactured by the Abbott Diagnostics used for the rapid qualitative detection of Histidine-rich Protein II (HRP-II) antigen and Lactose dehydrogenase (pLDH) from malaria Plasmodium falciparum. Studies have shown that this test kit has relatively good sensitivity/specificity compared to traditional testing methods like microscopy and polymerase chain reaction (PCR) [28, 29]. Therefore, clinicians need to test with RDTs when available instead of assuming they don’t give accurate results” |
|  | Also elaborate on ACTs (and other treatments provided. | We have revised the background to include information on ACTs used in health facilities in Ghana according to the national malaria treatment guidelines. This can be found on lines 90-97. |
|  | The figures are redundant and can be sumarised in the main body of the text | Thank you for the comment.  We have removed the figures and summarized them in the main text. |
| **Reviewer 2** | As the title of the manuscript describe, the authors have assessed the performance and challenges in the implementation a strategy called T3 (test-treat-track) for the control of malaria among children under 5 years in Ghana. No general comment to do. | Thank you for your brief overview of the manuscript |
|  | Specific comments: Abstract: Results: Line39-40: “Most children, 818/900 (90.9%) were tested for malaria and 600/818 (73.4%) were positive for malaria parasitaemia.” Which test was used detect malaria parasitemia? RDT or microscopy? Authors need to specify. | Thank you for your comment.  We have revised the results in the abstract to indicate the test that was used. This can be found on line 38-39 and reads as “Most children, 818/900 (90.9%) were tested for malaria and 600/818 (73.4%) were positive for malaria parasitaemia using rapid diagnostic test” |
|  | Line 43: “ Children completed T3 with CHPS compound “. Please give the explanation of this abbreviation. | We have written this term now in full and the sentence now reads as “Only 408/900 (45.3%) children completed T3 with Community Health-based Planning Services (CHPS) compound having the highest completion rate 202/314 (64.3%)” This can be found on line 42. |
|  | Introduction Line 63: “challenges of T3 strategy”. It the first time to use T3 in the main text. Please explain it. | Thank you for the comment. Based on another comment, where you suggested that we revise the introduction to make it shorter, we have deleted the portion that had this statement. |
|  | General comment: 3 page for the introduction is too long. I will ask authors to summary the introduction 1 page and half maximum. Some paragraphs can be summarized in few lines to make it consistent. I encourage the authors to review the introduction. | Thank you for your comment. We have revised the introduction and reduced it significantly. |
|  | Materials and methods Study site description Line 128-134 “the region are two ………. mountainous”. This section is not relevant for the reader. Maybe use a map to show the study site  Line 137-139: Not relevant. Reader want information that could be helpful for the reader to understand your study and data. General comment: There are more information that are not relevant for the ready. I will suggest the authors to summarize and give the relevant ones. | Thank you for your comment.  We have revised the study area to take out most of the descriptive information and used a map to show the municipalities/districts in which the study was conducted. The map is labelled as Fig 1. |
|  | Study population Line 146: “The second study “. Is it a cross-section survey or another study different to the main study? I think after enrolment of febrile children, cross-sectional survey were made to collect qualitative information. This should be clarify. | The study was a cross-sectional study that involved two different populations. The first population was caregivers of children under 5 years with a history of fever who were interviewed to find out if they had been tested for and or treated for malaria. The second population was clinicians assigned the duties of prescribing during the study period and were available. They were interviewed to determine the challenges hindering the implementation of the T3 strategy in health facilities.  The study population in the manuscript has been revised to make it clearer. This can be found on lines 169-172 and reads “The study had two different populations: (i) children under five years with their caregivers who reported to the health facilities with fever or history of fever and were managed by clinicians and (ii) clinicians rendering services at the various health facilities during the time of the study” |
|  | Study design I note that no biological data has been collected. Only interview of healthcare workers or care givers. | Thank you for this observation.  One of the objectives of the study was to assess the implementation of the T3 strategy as part of routine care in health facilities. That is why we interviewed care givers of children under 5 years who had accessed routine care at the health facilities to find out if the T3 strategy was followed. The use of exit interviews made it difficult to collect biological data such as verifying the test result written in the folder of the child with what was recorded in the OPD or laboratory register.  The other objective was to determine the challenges faced by clinicians in the implementation of the T3 strategy in health facilities, hence there was no need for biological data to answer this objective. |
|  | Results Prefer to not make comment. | Thank you |
|  | Discussion The discussion is made in 4 pages and half. In addition, In addition, the study design is not strong to make a solid analysis. Data collected are main based on information collected from care givers and healthcare workers. This has been noted by the authors and I think this is a major bias. | Thank you for the comment.  We have revised the discussion to shorten the length.  However, we politely disagree with the author that our study design was not strong to make solid analysis. The study design used enabled us to answer all the objectives of the study and our findings are relevant to the Ghana National Malaria Control Programme in identifying aspects of the T3 strategy that needs improvement.  We did state in our limitations that the use of cross-sectional study design made it impossible to determine seasonal variations of malaria but findings from this study are very relevant as stated above. |
|  | For me this paper cannot be accepted for publication. | Thank you, we appreciate your comment but we believe the revisions we have made to the manuscript based on the valuable comments you have given has improved the quality of the manuscript and it can be considered for publication |
| **Academic Editor** | it is also noted that samples were collected a considerable time ago. Please address this issue in your discussion. Also elaborate on the current validity of your observations | Thank you for your comment. We agree that the data was collected 5 years ago. In response to an earlier comment by reviewer 1 above, we have explained this point and included it in the discussion section of the revised manuscript.  This can be found on lines 459-462 and reads “Although data for this study was collected about 5 years ago, the findings are still relevant in informing interventions to improve adherence to the T3 strategy in health facilities in Ghana since to the best of our knowledge no specific intervention has been implemented in the regions the study was conducted to improve adherence to the strategy.” |
