# Supplementary material for: Assessment of the performance and challenges in the implementation of the test, treat and track (T3) strategy for malaria control among children under-five years in Ghana
Source: PLoS One. 2022 Dec 7;17(12):e0278602. doi: 10.1371/journal.pone.0278602 (PMC9728892; doi:10.1371/journal.pone.0278602)
Supplement: Attachment — Submitted filename: Response to Reviewers.docx [file pone.0278602.s003.docx]

**Response to Reviewers’ Comments**

| **Reviewer** | **Comment** | **Response** |
| --- | --- | --- |
| **Academic Editor** | Please review your reference list to ensure that it is complete and correct. If you have cited papers that have been retracted, please include the rationale for doing so in the manuscript text, or remove these references and replace them with relevant current references. Any changes to the reference list should be mentioned in the rebuttal letter that accompanies your revised manuscript. If you need to cite a retracted article, indicate the article’s retracted status in the References list and also include a citation and full reference for the retraction notice | Thank you for the comment.  We have reviewed our reference list and updated reference 8 which was incomplete. We have also updated the URL for reference 15 which initially was inaccurate. These changes can be found on page 25, line 450 and page 26, line 479.  We found none of our papers cited to have been retracted. |
| **Reviewer 1** | I think that the authors have sufficiently addressed my concerns. However, to be in line with PLOS ONE policy, there must be statements on access to data (now only a minimal data set has been made available) and ethics should be addressed | Thank you accepting the revisions we have made based on the comments you provided to improve the manuscript.  Access to data: we have provided the minimum dataset as a supporting information which is one of the acceptable data sharing methods for Plos One as per their data availability requirements. The data set provided contains all that is required to replicate all the study findings reported in the manuscript. We added a data availability statement under the data availability section of the online submission portal and the statement reads as “All relevant data are within the manuscript and its supporting information files”.  Although the preferred option is to deposit the data in a data repository, unfortunately our institution has no data repository for us to deposit our dataset. Also, this study did not receive any funding so we are unable to pay for the data to be deposited in a public data repository.  Ethics: we have a sub section titled ethical issues under the methods section of our manuscript where we have stated the ethics review boards that approved the study and the ethics approval numbers. We have revised the section to include the date of review and it reads as “Ethical clearance was obtained in May 2017 from the Ghana Health Service (GHS) Ethics Review Committee (ERC) (GHS-ERC) and the Dodowa Health Research Center (DHRC) Institutional Review Board (IRB) (DHRCIRB) with study approval numbers of GHS-ERC: 44/05/17 and DHRCIRB/25/05/17 respectively before the commencement of the study. Also, permission from the Municipal/District Health Directorates and the Health facilities was sought. A written informed consent was obtained from the (mother/guardian) of respondents as well as clinicians before commencement of the study”. This can be found on page 11, lines 216-223 of the revised manuscript. |
| **Reviewer 2** | No comment | Thank you for your comments that helped improve the manuscript. |
